# OCEAN project

**DOI:** 10.1002/alz70860_102355

**Published:** 2025-12-23

**Authors:** Michelle C Carlson, Kyle D. Moored, Omar Ahmad, Arnold Bakker, Emily Richards, Kylie H. Alm, Promit Roy, John Krakauer, Matthew E. Peters

**Affiliations:** ^1^ Johns Hopkins Bloomberg School of Public Health, Baltimore, MD, USA; ^2^ Neuroanimation incorporated, Columbia Falls, MT, USA; ^3^ Johns Hopkins University School of Medicine, Baltimore, MD, USA; ^4^ Neuroanimation Incorporated, Baltimore, MD, USA; ^5^ Johns Hopkins School of Medicine, Baltimore, MD, USA

## Abstract

**Background:**

This study seeks to address the feasibility of a novel Traumatic Brain Injury (TBI) intervention optimized for adults with a remote history of TBI by determining the short‐term impact on brain, mood, body, lifestyle activity and cognition, of an immersive 3‐dimensional (3‐D) computer game. The game requires one to help a dolphin, Bandit, through the ocean to catch fish and avoid getting bitten by sharks, thereby exercising and integrating complex cognitive‐motor processes for pro‐social goal. Here, we focus on whether this activity impacted subregions of the hippocampus related to verbal and spatial memory formation.

**Methods:**

During this 3‐month randomized, controlled trial (RCT), we compared weekly performance on a complex cognitive‐motor game, called “Bandit” (intervention) to a Successful Aging Program (active control) in those with history of TBI on Alzheimer's Disease Related Dementias ‐related brain biomarkers, and specifically, high‐resolution hippocampal volumetry of subregions.

**Results:**

A total of 23 participants were enrolled, with a mean age of 60.3 years in the intervention group and 57.6 years in the control group, 14 completed a brain MRI at baseline and 8 at follow‐up. Of these, There were no statistically significant group differences in age, sex, education, or race. There was higher MRI‐specific drop‐out at follow‐up in the active control condition (*N* = 4) vs. Bandit intervention group (*N* = 2). Participants in the Bandit intervention enjoyed the game and reported no adverse effects. Pre‐post MRI results showed significant intervention‐specific increases in volumes in the left subiculum (*p* = 0.043) and CA3 subregions (*p* = 0.020) of the hippocampus, both of which are related to spatial and verbal memory and risk for ADRD. No significant differences were observed in the left CA1, CA2, or dentage gyrus or any right subregions.

**Conclusions:**

Our results confirm that an immersive game involving complex motor activities for pro‐social benefit was enjoyed and well tolerated. Gamers with a history of TBI demonstrated improved subicular and CA3 volumes of the left hippocampus relative to active controls. These promising results have implications for early intervention designed for social, physical and cognitive engagement in single player and group player settings among those at risk for mild cognitive impairment and ADRD.

